# The Onus of Helping Necessitous Nurses

**Published:** 1921-07-09

**Authors:** 


### THE MATRONS' AND SISTERS' DEPARTMENT.

### THE ONUS OF HELPING NECESSITOUS NURSES.

Few nurses realise the energy and self-sacrifice which have been needed to secure the Endowment Fund of £100,000 for the nation's tribute to its nurses. Fewer still realise that this capital is still insufficient to meet the needs of the nurses who have fallen into distress through no fault of their own.

There are three main sources from which distress among nurses may be seen to arise. The first is the neglect in the past of any adequate provision for pensioning aged nurses or assisting such as fall into ill-health. An enormous bill of arrears has to be met by the administrators of the Nation's Tribute in respect of nurses who for years past have needed help without succeeding in obtaining it from any of the small existing funds for helping unattached nurses. Secondly, there is the wide range of modern nurses who have not succeeded in saving sufficient to procure themselves an independence in old age. The lack of any widespread scheme for the superannuation of nurses leads to many and great evils. Nurses cannot be said to have neglected their own duty in this respect. The growth and prosperity of the Royal National Pension Fund for Nurses testify to the existence of a large and thrifty section of the profession who are fully alive to the advantages of the saving habit and to the number of nearly 3,000 are now enjoying the fruit of their prudence and are collectively receiving at the rate of over £76,000 a year. But, regarded from the point of view of the entire profession, the number of nurses who are provided for by annuities bought out of their own savings is small contrasted with the number who if superannuation were once obtained would come under such a scheme. The number of nurses in the Pension Fund whose annuities began to fall in in last December was 209. How many new nurses would be eligible every year under any national schemes of superannuation it is impossible to estimate, but it would certainly run into four figures.

Lastly, the casualties of the nursing profession, the disabling illnesses, the misfortunes and fluctuation of a particularly exacting profession, constitute an unfailing source of distress, and create year by year new cases for temporary relief. Every nursing society which starts out to relieve its members in difficulties discovers the extent to which demands are made on its funds by self-respecting and independent women against their will. The Jubilee Institute, for instance, reports that last year the demands made on the Tate (Sick) Fund were exceptionally heavy, and grants were made amounting to £269. The story of the Junius S. Morgan Benevolent Fund is an illuminating commentary on the vicissitudes in nurses' lives. This fund, prior to the formation of the Nation's Tribute, was by far the largest available for the relief of nurses in distress. Its investments total £21,758; the grants made amounted last year to £2,084. It is reserved solely for the benefit of members of the Royal National Pension Fund—that is to say, for the most prosperous and thrifty nurses in the profession. Yet year by year the applications for aid are far too numerous to be dealt with out of the available income. Let those who vainly try to pretend that nurses are sufficient for themselves weigh well these figures.

There is one more word to be said. Depreciatory remarks on the generous assistance procured for nurses through the Tribute Fund are usually supported by easy assurances that nurses are perfectly well able not only to look after themselves but also to relieve any cases of distress which may arise in their own profession. These statements can easily be put to the proof. The committee of the Junius S. Morgan Fund were compelled last year to meet a deficit of £639, which was necessarily withdrawn from their invested funds. All that is necessary to met the cost of giving adequate relief to the members of the Pension Fund is the payment of one shilling a year by each member. This, we are told, would suffice to give an income of £500 a year. Yet out of the 10,000 members the number of those who took so much thought for their suffering sisters as to buy a shilling postal order or add this paltry amount to their annual premiums amounted to the magnificent total of 800, and the sum received was £40. Imagine being told that by the gift of one shilling a year you could meet all the calls for aid made by your fellow workers in distress and then failing to respond! Can there be a more glaring illustration of the imperative need for the Tribute Fund, no less than for compulsory superannuation?

